# Bioactive Betulin and PEG Based Polyanhydrides for Use in Drug Delivery Systems

**DOI:** 10.3390/ijms22031090

**Published:** 2021-01-22

**Authors:** Daria Niewolik, Barbara Bednarczyk-Cwynar, Piotr Ruszkowski, Tomasz R. Sosnowski, Katarzyna Jaszcz

**Affiliations:** 1 Department of Physical Chemistry and Technology of Polymers, Silesian University of Technology, M. Strzody 9, 44-100 Gliwice, Poland; Katarzyna.Jaszcz@polsl.pl; 2 Department of Organic Chemistry, Poznan University of Medical Science, Grunwaldzka 6, 60-780 Poznan, Poland; bcwynar@ump.edu.pl; 3 Department of Pharmacology, Poznan University of Medical Science, Rokietnicka 5a, 60-806 Poznan, Poland; pruszkowski@gmail.com; 4 Faculty of Chemical and Process Engineering, Warsaw University of Technology, Warynskiego 1, 00-645 Warsaw, Poland; tomasz.sosnowski@pw.edu.pl

**Keywords:** betulin, polyanhydrides, biodegradable microspheres, cytostatic activity, drug delivery systems

## Abstract

In the course of this study, a series of novel, biodegradable polyanhydrides based on betulin disuccinate and dicarboxylic derivatives of poly(ethylene glycol) were prepared by two-step polycondensation. These copolymers can be used as carriers in drug delivery systems, in the form of microspheres. Betulin and its derivatives exhibit a broad spectrum of biological activity, including cytotoxic activity, which makes them promising substances for use as therapeutic agents. Microspheres that were prepared from betulin based polyanhydrides show promising properties for use in application in drug delivery systems, including inhalation systems. The obtained copolymers release the active substance—betulin disuccinate—as a result of hydrolysis under physiological conditions. The use of a poly(ethylene glycol) derivative as a co-monomer increases the solubility and bioavailability of the obtained compounds. Microspheres with diameters in the range of 0.5–25 µm were prepared by emulsion solvent evaporation method and their physicochemical and aerodynamic properties were analyzed. The morphological characteristics of the microspheres depended on the presence of poly(ethylene glycol) (PEG) segment within the structure of polyanhydrides. The porosity of the particles depended on the amount and molecular weight of the PEG used and also on the speed of homogenization. The most porous particles were obtained from polyanhydrides containing 20% wt. of PEG 600 by using a homogenization speed of 18,000 rpm.

## 1. Introduction

In recent years, there has been growing interest in compounds that were obtained from renewable sources. Studies are being carried out on the isolation of substances from plant extracts that may be usable in, among others, cosmetics and pharmaceutical industries. Such compounds can be used directly as ingredients of preparations, but they may also be subjected to various modifications in order to obtain new compounds, including polymers. Betulin is one of such natural compounds.

Betulin is a pentacyclic triterpene that is obtained in large quantities from birch bark. Betulin and its derivatives exhibit a broad spectrum of biological activity [1]. Modification of the structure of betulin in C-3 and C-28 positions makes it possible to obtain derivatives with anti-inflammatory [2], anti-viral [3], and anti-cancer activity [1]. Thanks to those properties, the modified compounds are promising as new therapeutic agents. There are only several literature reports describing the polymeric forms of betulin, including polyesters [4], polyurethanes [5], and polyethylene oxide conjugates [6]. However, the majority of those polymers are used in non-biological applications, e.g., gas adsorption studies. Dicarboxylic derivatives of betulin, such as disuccinate betulin (DBB), are an excellent raw material for obtaining polyanhydrides. Disuccinate betulin exhibits broad biological activity, including anti-cancer, anti-leishmanic [7], hypolipidemic [8], fungicidal [9], bactericidal [10], and antiviral effects, including Epstein–Barr virus [3] and HIV [11].

Polyanhydrides are a class of surface-degradable polymers obtained by polycondensation of compounds containing two carboxylic groups. Polyanhydrides undergo hydrolytic degradation to their respective diacids that are completely eliminated from the body within a short period of time. Because of their lack of toxicity and appropriate release kinetics of active substances, they are used in medicine, as both drug carriers and biomaterials [12]. Recently, we obtained polyanhydrides of betulin disuccinate which exhibited anticancer activity and they can be used in drug delivery systems [13]. Polyanhydrides obtained from disuccinate betulin can be used as a polymeric prodrug, since the active substance (DBB) is chemically bound to the polymer chain and, by undergoing hydrolysis under physiological conditions, is gradually released into the organism. Betulin and betulin disuccinate both show a lack of toxicity in vitro and in vivo. In order to manufacture betulin-based copolymers in a form suitable for controlled drug release, attempts were made in order to obtain polymer microspheres.

Microspheres are a useful form of drug delivery system, because they can be used to encapsulate, protect, and control the release of a wide range of drugs. They can be easily administered either orally, in the form of injections [14], or as inhalable pulmonary systems. When delivered with use of microspheres system, toxic and insoluble drugs can be administered with a lower frequency and smaller quantity [15]. Microspheres can be obtained from a range of materials, including biodegradable polymers. The advantage of using biodegradable polymers is their ease of removal of the degradation products from the organism, due to their low molecular weight. Furthermore, by controlling the rate of polymer degradation, it is possible to manipulate the rate of release of active substances [14,16]. Polyanhydrides are an excellent material for the preparation of such microspheres, to their degradation and release characteristics. Because of the fact that polyanhydrides undergo a gradual hydrolytic degradation starting from the surface, they are expected to have a beneficial effect on the release profile of active substances. Microspheres have been investigated for use in the controlled release of drugs, such as chemotherapeutic agents, local anesthetics, anticoagulants, neuroactive drugs, and anticancer agents [12]. They can also offer an interesting alternative for oral and parenteral drug delivery, in the form of inhalation systems.

In modern pharmaceutical practice, drugs are administered via a pulmonary route for two main purposes: local or systemic therapies [16]. Locally acting drugs for inhalation are mainly bronchodilators, corticosteroids, and antibiotics [17,18,19,20,21]. Inhalable innovative drug delivery systems, such as microspheres, are expected to reduce the direct contact of highly concentrated drug formulations with the lung tissue and minimize the fluctuation of drug concentration due to sustained release [21]. Inhaled products administered for their systemic effects mainly include fast-onset analgesics, peptides, or proteins, which would otherwise need to be given in form of an injection [22,23,24,25]. The pulmonary delivery route has been attractive for systemic drug administration, due to the large surface area, high permeability, and slow mucociliary clearance of lung tissues. In order to use microspheres in inhalation systems, particles must have appropriate aerodynamic diameter. Particles that are below 5 µm can be distributed into smaller airways, while the particles with aerodynamic diameter in the 1–2 µm range are the most efficient for deposition into the capillary-rich alveolar airspaces [15,26]. The microspheres prepared from the DBB-based polyanhydrides have a unique potential for use as inhalation systems in cancer treatment, since loading them with chemotherapeutic agents can lead to a synergistic therapeutic effect.

The aim of this work was the synthesis and characterization of polyanhydrides that are composed of disuccinate betulin and dicarboxylic derivatives of poly(ethylene glycol), and the creation of microspheres from those materials, which could be used as drug carriers in controlled drug delivery systems. Hydrolytic degradation of copolymers and their cytostatic activity were investigated in order to use these polymers in pharmaceutical industry. The aerodynamic properties of obtained particles, which affect their suitability for use in inhalation system, such as particle size distribution, aerodynamic diameter, and fine particle fraction, were also examined.

## 2. Results and Discussion

### 2.1. Polyanhydrides

**Figure 1:**
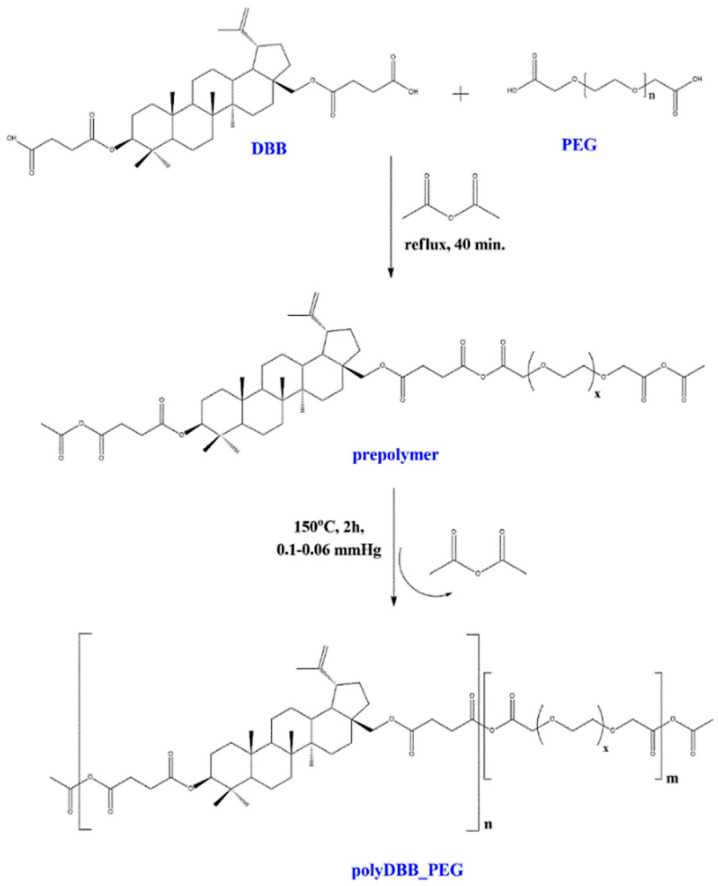
Reaction scheme of the synthesis of polyanhydrides based on disuccinate betulin (DBB) and dicarboxylic derivatives of PEG.

The polyanhydrides were obtained by melt polycondensation of betulin disuccinate and dicarboxylic derivatives of poly(ethylene glycol) (PEG) with the use of acetic anhydride (Figure 1). Betulin disuccinate (DBB) was synthesized by the esterification of succinic anhydride and betulin, following the procedure described earlier [13]. During previous work, we have obtained a betulin disuccinate homopolymer (polyDBB) [13], which exhibited anticancer activity. However, polyDBB degrades relatively slowly due to its strong hydrophobicity. The cytostatic effects of polyDBB can be modified by copolymerization of DBB with different diacids (e.g., dicarboxylic derivatives of poly(ethylene glycol), allowing for optimization of the degradation rate and thus, for a tailored period of cytotoxicity. In this study, two carboxylic derivatives of PEG with molecular weights of 250 and 600 were selected as co-monomers. PEG content in the synthesized copolymers ranged from 20% to 80%. The design intent was to retain DBB’s antitumor activity in the copolymer, while using the inclusion of the PEG derived co-monomers in order to increase the solubility and bioavailability of the obtained compounds. The physicochemical properties of obtained copolymers were thoroughly evaluated by a number of analytical and spectroscopic techniques.

**Figure 2:**
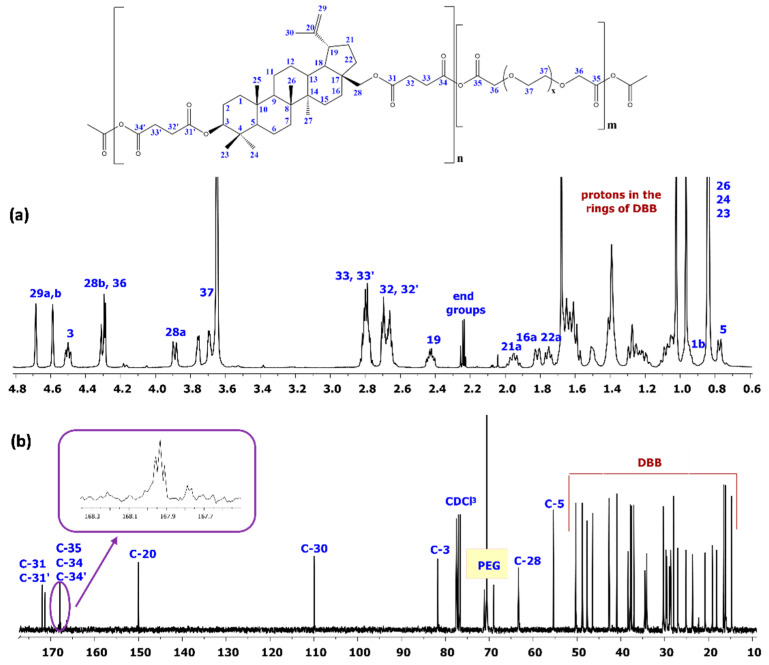
^1^H NMR and ^13^C NMR spectra of polyanhydrides based on betulin disuccinate and dicarboxylic derivatives of PEG. (**a**) ^1^H NMR spectrum; (**b**) ^13^C NMR spectrum.

The obtained polyanhydrides were solid, amorphous materials. FT-IR, ^1^H NMR, and ^13^C NMR spectra were obtained in order to determine their structure. The formation of anhydride bonds, and thus a successful synthesis of polyanhydrides was confirmed by the presence of two characteristic peaks in the carbonyl region of the FT-IR spectra at 1724 cm^−1^ and 1827 cm^−1^. Figure 2 shows the typical ^1^H NMR and ^13^C NMR spectra for the copolymers.

In the ^1^H NMR spectra, the signals at δ = 2.82–2.78 ppm (C_33_–H_2_, C_33′_–H_2_) and at δ = 4.30 ppm(C_36_–H_2_) are visible, which could be assigned to methylene protons that are close to anhydride groups in DBB and PEG part, respectively. The presence of those signals in ^1^H NMR spectrum further confirmed the formation of the polyanhydride.

The ^13^C NMR spectra of the copolymers showed signals that were assigned to carbonyl carbon atoms in anhydride (δ = 167.94 ppm; C_34_, C_34′_ and C_35_) and ester groups (δ = 171.95 ppm; C_31_ and C_31′_) as well as two different signals of methylene carbon atoms next to ester (δ = 28.89 ppm and δ = 28.89 ppm; C_32_ and C_32′_) and anhydride (δ = 30.35–30.18 ppm; C_33_ and C_33′_) groups, respectively.

In the ^1^H NMR and ^13^C NMR spectra, besides the signals confirming the presence of ester and anhydride bonds, signals that were attributed to methylene protons or methylene carbon atoms present in the repeating unit of PEG were also observed. The presence of the signals at δ = 4.30 ppm (C_37_–H_2_) in ^1^H NMR and δ = 71.12–70.56 ppm (C_37_) in ^13^C NMR spectra confirms presence of the repeating unit of PEG in polyanhydrides.

The rest of the ^1^H NMR and ^13^C NMR signals were assigned to the relevant polyDBB_PEG protons and carbons based on our previous work [13].

^1^H NMR was also used in order to determine the PEG and DBB content in copolymers, as well as the molecular weight of the copolymers. The DBB:PEG ratios and molecular weights of copolymers were calculated, according to Equations (1)–(6), while using the integration of the signals of protons that were assigned to DBB (δ = 4.68 and 4.59 (C_29_–H_a,b_), δ = 4.50 ppm (C_3_–H), δ = 4.30 ppm (C_28_–H_a_)), and PEG (δ = 4.30 ppm –CH_2_C(O)OC(O)– in PEG), as well as the signals of methylene protons near the anhydride groups (δ = 2.82–2.77 ppm (I_SAc_)), methylene protons near the ester groups (δ = 2.74–2.64 ppm (I_E_)), and the protons of end groups (δ = 2.24 ppm). The intensities of signals used to calculate the molecular weights of the polyanhydrides are summarized in the Tables S1–S8.

Table 1 summarizes the molecular weight values of copolymers calculated from ^1^H NMR and determined by gel-permeation chromatography (GPC).

**Figure 3:**
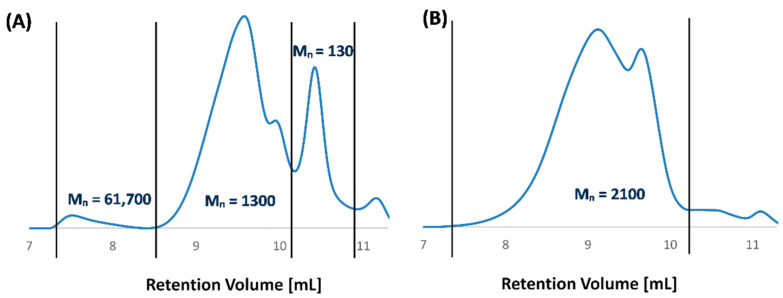
Gel-permeation chromatography (GPC) chromatograms of polyDBB_PEG_250_60 (**A**) and polyDBB_PEG_600_60 (**B**).

The molecular weight of polyanhydrides determined by GPC were much lower than the Mn that was calculated from ^1^H NMR spectra, because their hydrolytic instability resulted in a decrease in the polymer molecular weight during the preparation of the solutions and measurements. For copolymers containing PEG 250, the presence of a higher molecular fraction can be observed (Figure 3A). It is not a monomodal distribution. The GPC chromatogram of most polyanhydrides exhibited bimodal character (Figure 3) and indicated the presence of low molecular fraction, probably due to degradation products.

As an example, for the polymer containing 60 wt.% of PEG 600, higher molecular fractions were not observed, due to the rapid degradation of the polymer (Figure 3B). The comparatively slower degradation of polyDBB_PEG_250_60 (investigated in further detail in Section 2.2) is the likely cause for the presence of the small amount of high molecular mass (Mn > 60,000, Figure 3A). An analogous explanation is likely the cause of the Mn value discrepancies between ^1^H NMR and GPC methods that are visible in Table 1.

The content of PEG in copolymers, as calculated from ^1^H NMR, was slightly lower than the content of PEG in the reaction mixture, probably due to the hydrolytic degradation of polymers.

The thermal properties of the copolymers were investigated while using the DSC method. The results (Table 1) indicated that the obtained polyanhydrides were completely amorphous. No crystallinity was observed within the temperature range of −60 to 250 °C. Similarly to the newly obtained copolymers, PolyDBB itself is also an amorphous polymer with a high value of glass temperature (Tg = 124 °C). Even a low PEG content in the copolymers reduces the Tg, as compared to polyDBB, which affects the physical characteristics of the polyanhydrides. The copolymers containing 20 wt.% of PEG retain Tg significantly above room temperature, despite a significant decrease from that of polyDBB. Therefore, like polyDBB, they are in a glass state in room temperature, which allows for an easier process of microspheres production. Increasing the PEG content above 20 wt.% further reduces the Tg value of the obtained copolymers, which results in an increasing of their elasticity. In the DSC thermographs of the copolymers containing 40 and 60 wt.% of PEG 250, two different glass temperatures were observed: one related to polyPEG 250 segments (Tg < −40 °C) and the second one related to polyDBB segments (Tg > 15 °C). In the case of samples with higher PEG 250 content (80 wt.%), a single Tg was found, with the value within the range of the two Tg’s recorded for the 40 and 60 wt.% PEG 250 samples.

Table 2 summarizes the solubility testing results for the polyDBB_PEG samples. Polyanhydrides were found to be insoluble in water, ethanol (except the copolymers containing 80 wt.% PEG), diethyl ether, and hexane, but they could be dissolved in methylene chloride, chloroform, toluene, and THF. The increase of PEG content in copolymers improved their solubility in acetone and DMSO.

### 2.2. Hydrolytic Degradation and Stability of Copolymers

**Figure 4:**
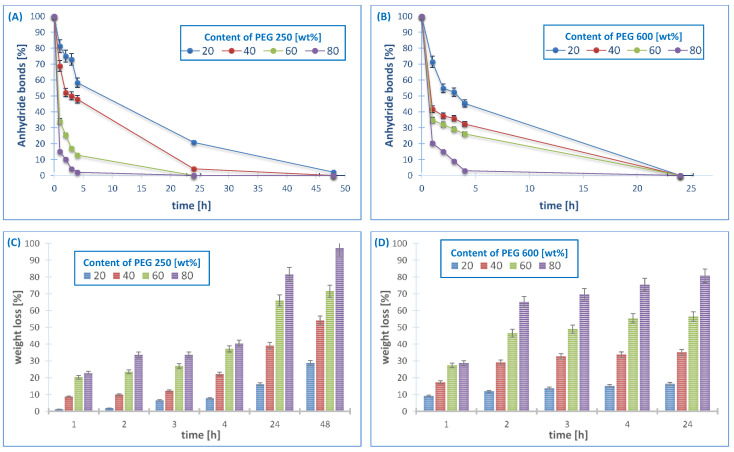
Anhydride bonds loss and weight loss of copolymers based on PEG 250 (**A**,**C**) and PEG 600 (**B**,**D**) during hydrolytic degradation in phosphate buffer conducted at 37 °C (*n* = 3, error bars, standard deviation).

The hydrolytic degradation was carried out in PBS (pH 7.4) at 37 °C in order to determine the disappearance of anhydride bonds and weight loss. The results of the experiment are shown in Figure 4. The ^1^H NMR spectra of the samples remaining after different degradation times revealed the presence of betulin disuccinate and dicarboxylic derivatives of PEG among the degradation products.

In the 1H NMR spectra of the degradation products, the signals at δ = 3.18 ppm, δ = 3.33 ppm and δ = 3.79 ppm that were assigned to methylene and methine protons next to hydroxyl groups were not observed. This signifies that no hydrolysis of the ester bonds in DBB took place, similarly to the degradation of polyDBB, as described in our earlier study [13].

It is known that the degradation rate of polyanhydrides is strongly dependent on the kind of diacid used in synthesis and it varies from a few days to several years [12,27,28,29]. For the obtained copolymers, the degradation rate is strongly dependent on the type and content of the hydrophilic component (PEG).

In our previous work [13], we showed the results of hydrolytic degradation of polyDBB. The homopolymer obtained from betulin disuccinate degraded relatively slowly due to its strong hydrophobicity. Under physiological conditions, it completely degrades in about 14 days. During this time, the complete disappearance of anhydride bonds in the sample was observed, but the weight loss of polyDBB sample was only about 20% (*w/w*) due to the poor solubility of degradation product (DBB) in PBS. However, the copolymers that are based on DBB and PEG degrade faster when compared to polyDBB, due to their increased hydrophilicity. The results shows that the degradation rate was slightly influenced by the molecular weights of comonomer (PEG), with a faster degradation of the PEG600-copolymers as compared to the PEG250 ones. However, with both of the comonomers, the degradation rate of the polymer increases strongly with the increased PEG content in the polyanhydrides. All of the copolymers containing PEG 600, due to their strong hydrophilicity, degraded completely in one day. During this time, the complete disappearance of anhydride bonds was observed and the weight loss of samples ranged from 20% to 90% (*w/w*).

**Figure 5:**
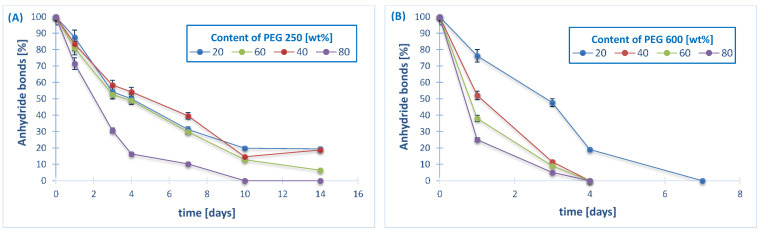
Anhydride bond loss of copolymers based on PEG 250 (**A**) and PEG 600 (**B**) in the air at 25 °C (*n* = 3, error bars, standard deviation).

The stability of the copolymers in air at room temperature was also investigated. The amount of anhydride bonds was determined after certain time of keeping the sample in air, as in the case of the hydrolytic degradation study (Figure 5).

The degradation rate in air increased with the increase of molecular weight of PEG, similarly to the degradation results in PBS. Polyanhydrides containing PEG 250 are more stable in air when compared to polyanhydrides containing PEG 600.The disappearance of anhydride bonds in the samples after 24 h was about 10–30%, for the copolymers containing PEG 250 and about 25–75% for the copolymers containing PEG 600. The copolymers obtained with an 80% PEG content degrade completely within a few days, 14 days for PEG 250 and four days for PEG 600, respectively. The disappearance of anhydride bonds in the samples after seven days was about 60–90% for the PEG250 samples (depending on the PEG content) and disappeared completely for all of the PEG600 samples.

### 2.3. Cytostatic Activity of Polyanhydrides

Polyanhydrides containing DBB and PEG were studied in order to determine their cytostatic activity against selected cancer cell lines. Cell lines representing cervix, breast, lung, liver, central nervous system, and nasopharynx tumors were used in these studies to find the concentrations causing inhibition of cell growth in culture by 50% (IC_50_). Table 3 lists the IC_50_ values obtained for DBB, polyDBB, and the copolymers. Human dermal fibroblasts (HDF) cells lines were used as non-proliferative cells, in order to compare the results from human cancer cell lines and establish the selectivity between cancer and non-cancer cells. Cytarabine was used as the internal standard for methodological evaluation and actinomycin D (widely used anticancer agent) was used in this experiment to compare with our compounds and their activity. Actinomycin D has at least two mechanism of action: the inhibition of topoisomerases (I and II), and DNA direct intercalation. Table 3 also includes the results that were obtained for monomer (DBB) and polyDBB, as described earlier [13].

The cytostatic tests indicated that obtained copolymers were effective in the inhibition of growth of cancer cells (IC_50_ < 20 µg mL^−1^), with limited cytotoxicity towards normal cells. The selectivity index, defined as the ratio of IC_50_ values between the normal and cancer cell lines (IC_50HDF_/IC_50cancer cell lines_), was the highest for PEG_600_20, with values as high as 3.05. Other tested copolymers, as well as DBB and polyDBB, had lower selectivity index, but it remained in the range of values between 1.5 and 2.08. IC_50_ values calculated for copolymers were lower than these determined for polyDBB, which is associated with the faster rate of hydrolytic degradation and the release of more betulin disuccinate in the duration of the experiment. The results shows that the cytostatic activity of the polyanhydrides based on betulin disuccinate can be manipulated by changing the kind of comonomer and its content, allowing for an optimization of the degradation rate and, thus, a tailored period of cytotoxicity, depending on the specific application. The polyanhydrides containing PEG 250 showed the highest cytostatic activity, however they were also cytotoxic towards normal cells and exhibited the lowest selectivity indexes among all of the tested samples. Poly(ethylene glycols) with molar mass below 400 Da can be toxic to humans, since they may undergo sequential oxidation into diacid and hydroxy acid metabolites by alcohol and aldehyde dehydrogenase [30]. This effect decreases with the increase in molecular weight of the molecule. For this reason, the use of PEG 600 proved to be a better candidate for increasing the cytostatic activity against cancer cells when introduced into the polymer in the right amount, without causing cytotoxicity toward normal cells. The copolymers that are based on betulin disuccinate, just like polyDBB, can be used as a degradation-based delivery systems for DBB or combined with other chemotherapeutic agents can lead to a synergistic therapeutic effect in cancer treatment.

### 2.4. Microspheres Preparation and Characterization

**Figure 6:**
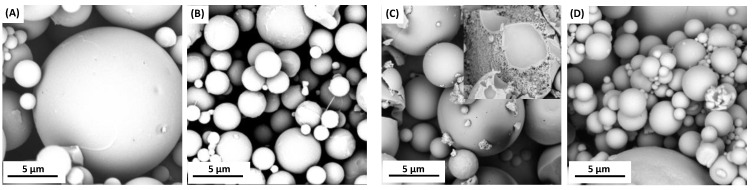
Scanning electron microscope (SEM) images of polyDBB_PEG_250_20 microspheres obtained by using homogenization speed of 3000 rpm (**A**) and 18,000 rpm (**B**) and polyDBB_PEG_250_40 microspheres obtained by using homogenization speeds of 3000 rpm (**C**) and 18,000 rpm (**D**).

**Figure 7:**
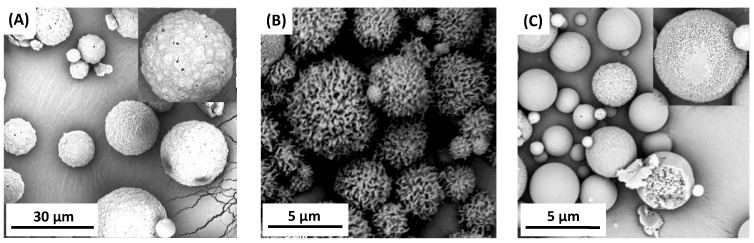
SEM images of polyDBB_PEG_600_20 microspheres obtained by using homogenization speed of 3000 rpm (**A**), 18,000 rpm (**B**) and 9000 rpm (**C**).

Attempts were made to obtain polymer microspheres in order to use the copolymers based on DBB and PEG in controlled drug delivery systems. Microspheres were prepared by an emulsion (O/W) solvent evaporation technique while using poly(vinyl alcohol) as stabilizing agent. By changing the speed of homogenization, it was possible to form smooth or porous particles with diameters of 3–30 µm (3000 rpm), 1–15 µm (9000 rpm), and 0.5–5 µm (18,000 rpm). The microspheres were prepared from two polyanhydrides with PEG 250 (containing 20 or 40 wt.%) (Figure 6) and from one polyanhydride with PEG 600 (containing 20 wt.% PEG) (Figure 7). The preparation of stable microspheres from remaining copolymers was impossible. A lack of crystallinity of such copolymers and their Tg being below room temperature resulted in a gluing of microspheres already in aqueous dispersion or after their separation. Additionally, copolymers containing a high amount of PEG undergo a faster hydrolytic degradation, which makes it difficult to obtain microspheres. Copolymer containing 40 wt.% of PEG 250 has a Tg value that is close to room temperature (Tg = 23.9 °C), which causes a sticking of the obtained particles, as observed on the SEM images (Figure 6C,D).

The microspheres that were obtained from copolymers containing PEG 250 had a smooth surface, but they were porous inside (internal porosity was observed for particles from copolymers containing 40 wt.% of PEG 250), while microspheres that were obtained from copolymer containing PEG 600 had a porous surface. The morphological characteristic of microspheres that were derived from polyDBB_PEG_600 was dependent on the speed of homogenization. Increasing the homogenization rate increases the porosity of the obtained microspheres. The most porous particles were obtained at a homogenization speed of 18,000 rpm. Based on obtained results, it can be concluded that the presence of PEG segment within the structure of polyanhydride could significantly alter the surface and internal morphology of microspheres. In addition, increasing the homogenization rate reduces the diameter and increases the porosity of the microspheres.

The size and size distribution of microspheres were calculated from optical microscope and SEM images (Table 4). D_n_ of microspheres calculated from SEM images were lower than those calculated from optical microscopic images. The obtained microspheres may be considered to be vehicles for biologically active compounds in drug delivery systems, including inhalation systems.

Polyanhydrides are hydrolytically degradable, which is why it was necessary to quantify the degree of hydrolytic degradation that occurs during the microsphere preparation. For this purpose, the anhydride bonds content was determined, based on ^1^H NMR spectra, for neat polymer (before microspheres fabrication) and for the polymer after the microspheres preparation. The disappearance of anhydride bonds was observed for all of the microspheres and dependent on the polymer composition and size of microparticles. For the microspheres with diameters of 5–20 µm (3000 rpm), a 5–10% disappearance of anhydride bonds, and for microspheres with diameter of 0.5–5 µm (9000 and 18,000 rpm), a 10–25% disappearance of anhydride bonds, were observed, after microparticles formulation. The degree of hydrolysis increased as the PEG content in copolymer increased. The process of hydrolytic degradation of polymers was more significant when small microspheres were produced. It was due to an increased surface area being available for contact with water. Such dependence is characteristic for polyanhydrides, which undergo hydrolytic degradation at the surface.

The porosity of the microspheres is a desirable property due to the fact that high porosity lowers the density and aerodynamic diameter of particles, and it improves the aerosol’s carrying capacity in the air stream, which affects the applicability of powder materials in inhalation systems. The use of large porous particles was first broadly introduced by Edwards et al. [31]. Porous or hollow particles with a density much lower than 1 g/cm^3^ and a geometric size larger than 5 µm, but with the aerodynamic diameter within the respirable size range, can be successfully used in pulmonary drug delivery systems [31,32,33]. When compared to smaller, non-porous micro and nanospheres, these particles are found to more efficiently disperse from a standard inhaler device. This is due to a reduction in the Van der Waals forces, which limits the particle interaction, aggregation, and coagulation [33]. Moreover, the large size of porous particles can resist macrophage clearance and allow a long therapeutic time for more controlled release [34].

In order to verify the usability of the obtained particles in inhalation drug delivery systems, all of the microspheres were characterized for their micrometric properties, such as: powder density (estimated as the tapped and untapped ones), aerodynamic diameter, Carr’s Index, and Hausner Ratio (Table 5).

The aerodynamic diameter (dae) is one of the most important parameters, because it has a critical impact on the deposition of inhaled preparation in the upper respiratory tract. Particles that are smaller than 5 µm can be distributed deep into the smaller airways, which is associated with a good clinical response to local treatment. The fraction of particles with an aerodynamic diameter in the range of 1–2 µm is probably the most effective for deposition in inhalation drug delivery systems. Aerodynamic diameter values were estimated on the basis of definition, according to Equation (5). The microspheres that were obtained by using homogenization speed of 18,000 rpm (aerodynamic diameter in the range of 1–2 μm) meet the conditions for use in inhalation drug delivery systems.

To obtain information regarding the flow properties of microspheres, the compressibility index (Carr’s index) and Hausner ratio (HR) (parameter characterizing flowability of powders) have been determined. These parameters are also important when analyzing the possibility of using the powder in inhalation drug delivery systems. Carr’s Index and Hausner Ratio only depend on the tapped and untapped density values. Microspheres that were obtained from polyDBB_PEG_250 displayed high values of Carr’s index (IC > 40) and Hausner Ratio (HR > 1.7), which indicates their extremely poor flow ability, while microspheres that were obtained from polyDBB_PEG_600 displayed better values of Carr’s (IC < 35) and Hausner Ratio (HR ≤ 1.5). The best results were obtained for polyDBB_PEG_600_20 microspheres that were obtained using homogenization speed of 18,000 rpm (passable flow ability). The appropriate flow and aerodynamic properties of these microspheres indicate their usability for inhalation drug delivery.

### 2.5. Aerosol Properties of Selected Microspheres

**Figure 8:**
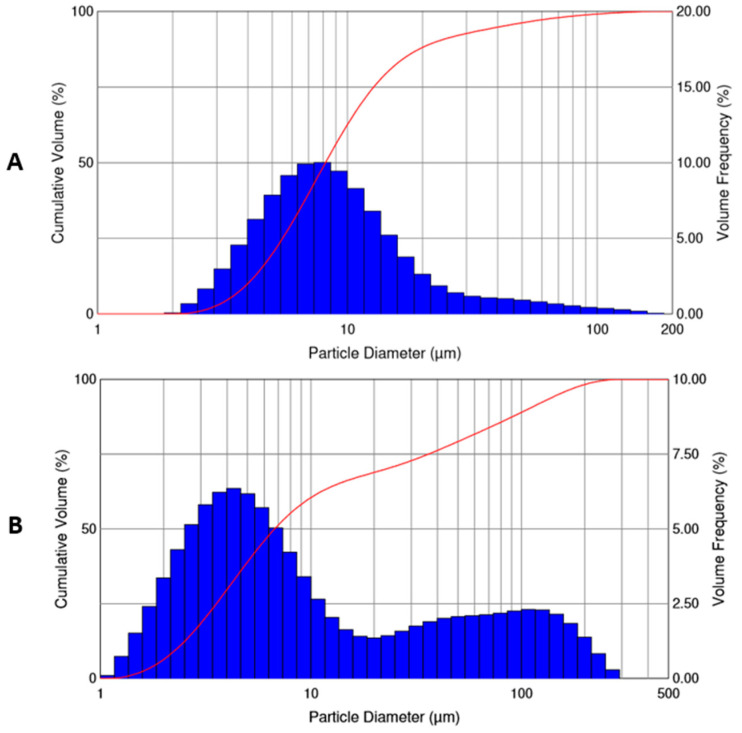
Volume size distribution of particle fraction in powder inhalers, polyDBB_PEG_600_20 microspheres, obtained by using homogenization speed of 9000 rpm (**A**) and 18,000 rpm (**B**).

PolyDBB_PEG_600_20 microspheres that were obtained by using homogenization speed of 9000 rpm and 18,000 rpm, (selected due to their favorable aerodynamic diameter and flow ability) were further tested for their aerosol properties. The powder aerosolization of microspheres was tested while using a capsule inhaler. Figure 8 shows the size distribution of particles dispersed in the air. The size distribution of the inhaled particles was used to evaluate the volumetric median diameter (VMD = D_V_50) and the percentage of particle fraction smaller than 5 µm (FPF) and 10 µm. The VMD and FPF are the most important parameters for the evaluation of aerosol suitability for drug delivery via inhalative route.

Table 6 presents the comparison of data that were obtained for these two types of microspheres. The comparison shows that the microspheres obtained by using a homogenization speed of 18,000 rpm are characterized by a slightly smaller median diameter (VMD) and have twice as large a value of FPF as compared with the microspheres obtained at lower homogenization speed. However, this powder has a broader particle size distribution, which can be seen from the data shown in Figure 8 and confirmed by the significantly higher span value.

The volume size distribution of aerosolized particles obtained while using a homogenization speed of 18,000 rpm has a bimodal character and shows the presence of two distinct fractions: one within the diameter in the desired size range (inhalable particles) and the second one with the diameter larger than 10 µm. The volume size distribution of particles that were obtained with use of homogenization speed of 9000 rpm is narrower, although, in this case, a small proportion of particles larger than 10 µm is also observed. Both of the tested powders have the same high content (above 60%) of inhalable particles (smaller than 10 µm). In spite of the discussed differences, both types of microspheres may be considered to be good candidates for carriers of inhaled drug. The presence of larger particles suggests a non-ideal microsphere deaggregation in the inhaler. Their aerosol properties can be improved by further manipulating the conditions of obtaining particles or sieving/separating them from particles of too large a diameter, which will reduce the proportion of aerosol particles with a size larger than 10 µm and will simultaneously increase FPF.

## 3. Materials and Methods

### 3.1. Materials

Betulin > 95% (Natchem), succinic anhydride 99% (Sigma-Aldrich, St. Louis, MO, USA), pyridine (min. 99.5%, Chempur, Karlsruhe, Germany), acetic anhydride (POCh S.A., Gliwice, Poland), dicarboxylic derivatives of poly(ethylene glycol) (with Mn = 250 and Mn = 600) (ACROS Organics, Fair Lawn, NJ, USA), DMSO (Chempur), trichloroacetic acid (Chempur), acetic acid (Chempur), Tris buffer, sulforhodamine, poly(vinyl alcohol) (M_w_ = 88,000 g/mol, 88% hydrolyzed) (ACROS Organics), and methylene chloride (Chempur) were used as supplied.

KB, HeLa, MCF-7, and Hep-G2 cells were obtained from the European Collection of Cell Culture (ECACC) supplied by Sigma Aldrich. A-549, U-87 and HDF cells were purchased from the American Type Culture Collection (ATCC) through LGC Standards (Lomianki, Poland). KB, Hep-G2, and U-87 cells were cultured in EMEM medium, while HeLa cells were grown in RPMI 1640 medium, A-549 cells in F-12K medium, MCF-7 cells in DMEM medium, and HDF cells in Fibroblasts Growth Medium. Each medium was supplemented with 10% fetal bovine serum, 1% l-glutamine and 1% penicillin/streptomycin solution. All of the cultures were maintained at 37 °C in a humidified atmosphere containing 5% CO_2_.

### 3.2. Synthesis of Disuccinate Betulin (DBB)

Disuccinate betulin was obtained according to the procedure described earlier [13] as a white, amorphous powder. Yield: 92%, m.p. 106–110 °C, ESI-MS m/z 665,4 [M + Na]^+^ (Calcd for DBB: 642.87).

IR: v = 2941cm^−1^ (m, v_C–H_), 2844 cm^−1^ (w, v_C–H_), 1724–1706 cm^−1^ (s, v_C=O_), 1310–1155 cm^−1^ (v_C–O_); 1603 cm^−1^, 982 cm^−1^, 880 cm^−1^(m, v_C=C_).

^1^H NMR (600 MHz, CDCl_3_, δ): 4.68 (1H, d, *J* = 1.7 Hz, C_29_–H_a_); 4.59 (1H, d, *J* = 0.2 Hz, C_29_–H_b_); 4.50 (1H, t, *J* = 7.9 Hz, C_3_–H_α_); 4.30 (1H, d, *J* = 11.0 Hz, C_28_–H_a_); 3.88 (1H, d, *J* = 11.0 Hz, C_28_–H_b_); 2.72–2.64 (6H, m, HOOC–CH_2_–CH_2_–COO–, –OOC–CH_2_–CH_2_–COOH); 2.64–2.58 (2H, m, HOOC–CH_2_–CH_2_–COO–); 2.42 (1H, dt, *J* = 5.7 and 10.3 Hz, C_19_–H); 1.68 (3H, s, C_30_–H_3_) 1.95 (1H, m, C_21_–H_a_); 1.81 (1H, d, C_16_–H_a_); 1.74 (1H, t, C_22_–H_a_); 1.69 (3H, s, C_30_–H_3_); 1.68–1.56 (7H, m, C_15_–H_a_, C_1_–H_a_, C_12_–H_a_, C_13_–H_a_, C_2_–H_a,b_, C_18_–H); 1.51 (1H, m, C_6_–H_a_); 1.45–1.35 (5H, m, C_6_–H_b_, C_11_–H_a_, C_21_–H_b_, C_7_–H_a,b_); 1.28 (1H, d, C_9_–H); 1.26–1.17 (2H, m, C_11_–H_b_, C_16_–H_b_); 1.13–1.04 (3H, m, C_22_–H_b_, C_12_–H_b_, C_15_–H_b_); 1.03 (3H, s, C_25_–H_3_); 0.97 (3H, s, C_27_–H_3_); 0.94–0.92 (1H, m, C_1_–H_b_); 0.86–0.82 (3 × 3H, 3 × s, C_26_–H_3_, C_23_–H_3_, C_24_–H_3)_; 0.78 (1H, d, C_5_–H).

^13^C NMR (150 MHz, CDCl_3_, δ): 178.0 (C_q_, C(O)OH); 172.37, 171.77 (C_q_, C(O)O); 150.09 (C_q_, C-20); 109.88 (CH_2,_ C-29); 81.55 (CH, C-3); 63.19 (CH_2,_ C-28), 55.39 (CH,C-5); 50.24 (CH, C-9); 48.76 (CH, C-18), 47.71 (CH, C-19); 46.42 (C_q_, C-17); 42.69 (C_q,_ C-14); 40.87 (C_q_, C-8); 38.34 (CH_2_, C-1); 37.82 (C_q_, C-10); 37.58 (CH, C-13); 37.04 (C_q_, C-4); 34.40 (CH_2_, C-22); 34.07 (CH_2_, C-7); 31.23 (CH_2_C(O)OH); 29.62 (CH_2_, C-21); 29.53 (CH_2_, C-16); 29.02 (CH_2_C(O)O); 27.88 (CH_3_, C-23); 27.00 (CH_2_, C-15); 25.13 (CH_2_, C-12); 23.58 (CH_2_, C-2); 20.80 (CH_2_, C-11); 19.08 (CH_3_, C-30); 18.15 (CH_2_, C-6); 16.51 (CH_3_, C-25); 16.14 (CH_3_, C-26); 16.02 (CH_3_, C-24); 14.80 (CH_3_, C-27).

DEPT: 6 × CH_3_, 16 × CH_2_, 6 × CH.

### 3.3. Prepolymers and Polymers Synthesis

The copolymers were obtained by a two-step melt polycondensation of betulin disuccinate and dicarboxylic derivatives of poly(ethylene glycol), according to the procedure described earlier [13,35]. Two dicarboxylic derivatives of PEG (with Mn = 250 and Mn = 600) were used in order to obtain the polyanhydrides. The betulin disuccinate and the derivatives of PEG, mixed in defined ratios (Table 7), were refluxed in acetic anhydride (1:10, *w/v*) under nitrogen flow for 40 min.

After this time, the excess of acetic anhydride and acetic acid formed in reaction was removed under vacuum. The remaining diacyl derivative of disuccinate betulin and PEG (prepolymer) was heated at 150 °C for 2 h with a constant stirring under vacuum (0.1 mm Hg) and nitrogen. The copolymers (polyDBB_PEG) in form of a solid, amorphous materials were obtained with a yield of over 90%. The obtained polymers were stored in a freezer.

IR: v = 2941cm^−1^ (m, v_C–H_), 2844 cm^−1^ (w, v_C–H_), 1827 cm^−1^ (m, v_C=O_), 1724 cm^−1^ (s, v_C=O_), 1034 cm^−1^ (v_C–O_).

^1^H NMR (600 MHz, CDCl_3_, δ): 4.68 (1H, d, C_29_–H_a_); 4.59 (1H, d, C_29_–H_b_), 4.50 (1H, t, C_3_–H_α_); 4.30 (1H, d, C_28_–H_a_ and CH_2_C(O)OC(O) in PEG); 3.89 (1H, d, C_28_–H_b_); 2.82–2.77 (4H, m, –OCOOC–CH_2_–CH_2_–COO–); 2.74–2.64 (4H, m, –OCOOC–CH_2_–CH_2_–COO–); 2.42 (1H, td, C_19_–H); 2.24 (end groups); 1.96 (1H, m, C_21_–H_a_); 1.82 (1H, d, C_16_–H_a_); 1.76 (1H, t, C_22_–H_a_); 1.68 (3H, s, C_30_–H_3_); 1.67–1.54 (7H, m, C_15_–H_a_, C_1_–H_a_, C_12_–H_a_, C_13_–H_a_, C_2_–H_a,b_, C_18_–H); 1.51(1H, m, C_6_–H_a_); 1.45–1.35 (5H, m, C_6_–H_b_, C_11_–H_a_, C_21_–H_b_, C_7_–H_a,b_); 1.29 (1H, d, C_9_–H); 1.26–1.17 (2H, m,C_11_–H_b_, C_16_–H_b_); 1.13–1.04 (3H, m, C_22_–H_b_, C_12_–H_b_, C_15_–H_b_); 1.02 (3H, s, C_25_–H_3_); 0.97 (3H, s, C_27_–H_3_); 0.94–0.92 (1H, m, C_1_–H_b_); 0.88–0.80 (9H, s, C_26_–H_3_, C_23_–H_3_, C_24_–H_3)_; 0.78 (1H, d, C_5_–H).

^13^C NMR (150 MHz, CDCl_3_, δ): 171.95 (C_q_, C(O)O); 167.94 (C_q_, C(O)OC(O)); 150.03 (C_q_, C-20); 110,0 (CH_2,_ C-29); 81.73 (CH, C-3); 71, 12–70, 56 ((–O–CH_2_–CH_2_–) in PEG); 63.32 (CH_2,_ C-28), 55.36 (CH,C-5); 50.25 (CH, C-9); 48.78 (CH, C-18), 47.68 (CH, C-19); 46.40 (C_q_, C-17); 42.68 (C_q,_ C-14); 40.88 (C_q_, C-8); 38.33 (CH_2_, C-1); 37.84 (C_q_, C-10); 37.58 (CH, C-13); 37.03 (C_q_, C-4); 34.48 (CH_2_, C-22); 34.07 (CH_2_, C-7); 30.35–30.18 (CH_2_C(O)OC(O)); 29.70 (CH_2_, C-21); 29.54 (CH_2_, C-16); 28,89 and 28.60 (CH_2_C(O)O); 27.95 (CH_3_, C-23); 27.0 (CH_2_, C-15); 25.13 (CH_2_, C-12); 23.62 (CH_2_, C-2); 20.78 (CH_2_, C-11); 19.12 (CH_3_, C-30); 18.13 (CH_2_, C-6); 16.51 (CH_3_, C-25); 16.13 (CH_3_, C-26); 16.0 (CH_3_, C-24); 14.74 (CH_3_, C-27).

### 3.4. Characterization of Polyanhydrides

The NMR spectra of polymers in CDCl_3_ were recorded on a Varian 600 MHz spectrometer with TMS as an internal standard. The infrared (FT-IR) spectra were recorded while using a PerkinElmer Spectrum Two Spectrometer. Molecular weights of polyanhydrides were determined in methylene chloride by gel-permeation chromatography (GPC) using Agilent Technologies Infinity 1260 chromatograph that was equipped with a refractive index detector and calibrated with polystyrene standards. The samples were pre-filtered before the analysis. Molecular weights were also calculated from the ^1^H NMR spectra, based on Equations (1)–(6).

(1) $$M_{w}=n_{DBB}M_{DBB}+n_{PEG}M_{PEG}+M_{T}$$

(2) $$I_{\left[1H\right]DBB}=\left(I_{C29-Ha}+I_{C29-Hb}+I_{C3-H}+I_{SAc}+I_{E}\right)/11$$

(3) $$I_{\left[1H\right]T}=I_{T}/6$$

(4) $$I_{\left[1H\right]PEG}=\left(I_{\delta =4.3\;\mathrm{ppm}}-I_{\left[1H\right]DBB}\right)/4$$

(5) $$n_{DBB}=\frac{I_{\left[1H\right]DBB}}{I_{\left[1H\right]T}}$$

(6) $$n_{PEG}=\frac{I_{\left[1H\right]PEG}}{I_{\left[1H\right]T}}$$

where: *M_DBB_*—molar mass of repeating unit of DBB in polyanhydride equal to 642.86 g/mol, *M_PEG_*—molar mass of repeating unit of PEG equal to 250 or 600 g/mol, *M_T_*—molar mass of terminal groups (end groups) in polyanhydride equal to 102 g/mol (molar mass of acetic anhydride), *I_*[1*H*]*DBB_*—intensity of one DBB proton, *I_*[1*H*]*PEG_*—intensity of one PEG proton, *I_*[1*H*]*T_*—intensity of one proton of end groups, *I_δ *= 4.30 ppm*_*—intensity of signal of five protons (C_28_–H_a_ and -CH_2_C(O)OC(O)- in PEG), *I_T_*—intensity of signal of terminal groups (*δ* = 2.24 ppm), *I_*(*C*29*-Ha*)*_* and *I_*(*C*29*-Hb*)*_*—intensity of signal assigned to methylene protons at the double-bonded carbon (*δ* = 4.68 and 4.59 ppm), *I_*(*C*3*–H*)*_*—intensity of signal assigned to metine proton in the ring of betulin (*δ* = 4.50 ppm), *I_Sac_*—intensity of signal assigned to methylene protons in the anhydride moiety (*δ* = 2.82–2.77 ppm), and *I_E_*—intensity of the signal assigned to methylene protons in the ester moiety (*δ* = 2.74–2.64 ppm).

The thermal analyses were performed while using a 822^e^ DSC Mettler Toledo differential scanning calorimeter. The samples were tested in temperature range from –60 °C to 250 °C at a heating rate of 10 °C/min.

### 3.5. Hydrolytic Degradation of Copolymers

The hydrolytic degradation experiments were performed in a phosphate buffer solution of pH 7.4 (PBS) at 37 °C. The hydrolytic degradation was monitored by recording both the mass loss of the test samples and the content of the anhydride groups. The solid samples of the copolymers (approximately 0.1 g) were placed in the weighed filters. Subsequently, the filters with copolymers were placed in glass vials containing 15 mL of PBS. The vials were incubated at 37 °C for a defined period of time (from 1 h to 14 days). After incubation, the buffer solutions were decanted. The remaining samples were rinsed with distilled water, dried to constant weight in a vacuum oven, weighted to the nearest 0.0001 g, and then tested using ^1^H NMR in order to calculate the content of anhydride groups. The mass loss was defined, as follows:

(7) $$\Delta m=\frac{m_{1}-m_{2}}{m_{1}}\times 100\%$$

where *m*_1_ represents the weight of the dry sample before degradation and *m*_2_ represents the weight of dry sample after degradation at the defined time interval.

The ratio of anhydride groups to the sum of anhydride and ester groups in the polyanhydride (*A*/*A* + *E*) was calculated while using the formula:

(8) $$\left(\frac{A}{A+E}\right)=\frac{I_{SAc}}{I_{SAc}+\;I_{E}}$$

where *I_SAc_* represents the intensity of proton signals of the methylene group in the anhydride moiety with a chemical shift (*δ*) of 2.82–2.77 ppm and I_E_ represents the intensity of the proton signals of the methylene group in the ester moiety with a chemical shift (*δ*) of 2.74–2.64 ppm.

### 3.6. Stability of Copolymers

The degradation rate of the copolymers in air at room temperature was also investigated. For this purpose, several samples (each weighing approximately 0.1 g) of each copolymer were placed in a glass vessel and left in the air (air humidity during the test was in the range of 40–50%). Every few days, one sample of each polymer was tested by ^1^H NMR, in order to calculate the content of anhydride moieties to the sum of the anhydride and ester moieties. Calculations were carried out in the same manner as in the case of hydrolytic degradation.

### 3.7. Cytostatic Activity of Polyanhydrides

The protein-staining sulforhodamine B (SRB) assay was employed for the determination of the cytotoxic activity of test compounds. The SRB method has been assessed by the National Cancer Institute (Bethesda, MD, USA) to be suitable for the in vitro anti-tumor screening [36]. The SRB assay estimates the cell densities based on the measurement of cellular protein content. For the SRB assay, 100 µL of a diluted cell suspension containing approximately 10^4^ cells was added to the wells of a 96-well plate. After 24 h, when a partial monolayer was formed, the supernatant was aspirated and 100 µL medium containing test compounds (DBB and polyDBB) at six different concentrations (0.1, 0.2, 1, 2, 10, and 20 µg/mL) was added to the cells. Stock solutions of test compounds were prepared in DMSO and the concentration of DMSO in the assay did not exceed 0.1%, which was found to be nontoxic to the applied cell lines. Following an incubation period of 72 h, 25 µL of 50% trichloroacetic acid was added to each well and the plates were incubated for 1 h at 4 °C. Subsequently, the plates were washed with distilled water in order to remove traces of the medium and then air-dried. When complete, the dried plates were stained with 100 µL 0.4% SRB (prepared in 1% acetic acid) for 30 min. at room temperature. Unbound dye was removed by rapid washing with 1% acetic acid and the plates were air-dried overnight. Finally, the protein-bound dye was dissolved in 100 µL of 10 mM unbuffered Tris and the absorbance was read at 490 nm.

### 3.8. Microspheres Preparation

The obtained polyanhydrides were used to obtain polymer microspheres. Microspheres were prepared by solvent evaporation from a polymer solution dispersed in aqueous phase, according to previous reports [13,35]. The polymer solution in methylene chloride (20 mL, concentration 50 mg/mL) was emulsified in 400 mL of aqueous solution (1% *w/w*) of poly(vinyl alcohol) (PVAl 88% hydrolyzed, MW = 88,000 g/mol), using ULTRA-TURRAX T18 homogenizer, for 30s. The speed of homogenizer was 3000, 9000 or 18,000 rpm, respectively. The emulsion was stirred with a magnetic stirrer at 1100 rpm at room temperature for 2.5 h to evaporate the organic solvent. After that microspheres were collected by centrifugation at 5000 rpm for 5 min., washed three times with distilled water, lyophilized and stored in freezer.

### 3.9. Characterization of Microspheres

The shape, size, and size distribution of particles were estimated using an optical microscope DELTA Optical ME 100 and PHAMIAS 2003 v.1.3 B software. The diameters of at least 100 particles were measured on each of the photomicrograph (magnification 100× and 400×). Subsequently, the number average diameters (*D_n_*) (Equation (9)) and volume average diameters (*D_v_*) (Equation (10)), standard deviation (S), and dispersity index (*D_v_*/*D_n_*) were calculated.

(9) $$D_{n}=\frac{\sum^{\;}N_{i}D_{i}}{\sum^{\;}N_{i}}$$

(10) $$D_{v}=\frac{\sum^{\;}N_{i}D_{i}^{4}}{\sum^{\;}N_{i}D_{i}^{3}}$$

where *N_i_* is the number of particles having diameter *D_i_.*

The morphological characterization of microspheres was carried out while using a Phenom ProX scanning electron microscope (SEM), after sputter coating of samples with gold.

In order to verify the usability of the obtained particles in inhalation drug delivery systems, all of the microspheres were characterized for their micrometric properties, such as: powder density (estimated as the tapped and untapped ones), compressibility index, flow and aerodynamic properties. Tapped density was estimated, as follows: microspheres were loaded into 0.3 mL section of a 1 mL plastic pipette that was capped with a teflon cap and tapped approximately 500 times until the volume of the powder did not change. Tapped density was calculated from the difference between the weight of the pipette before and after loading, divided by the volume of powder after tapping.

The aerodynamic diameter (*d_ae_*) of the particles (defining the mechanism of particle deposition in the respiratory system [37]) was estimated on the basis of definition, according to Equation (11).

(11) $$d_{ae}=d_{v}\sqrt{\frac{\rho_{T}}{X\rho_{0}}}$$

where *d_v_* is a volume-equivalent diameter, *ρ_T_* is tapped density, *ρ*_0_ is a reference density of 1 g/cm^3^, and *X* is the dynamic shape factor, which is 1 for a sphere [20,37].

To obtain information regarding the flow properties of microspheres, the compressibility index (Carr’s index) and Hausner ratio (HR) have been determined. Carr’s index was estimated through the relative percent difference between tapped and untapped density (Equation (12)), and the Hausner Ratio was estimated from the ratio between tapped and untapped density of the powder (Equation (13)) [38].

(12) $$IC=\frac{\rho_{T}-\rho_{UT}}{\rho_{UT}}\times 100\%$$

(13) $$HR=\frac{\rho_{T}}{\rho_{UT}}$$

where *ρ_UT_* and *ρ_T_* are untapped and tapped density of a powder, respectively.

Table 8 shows the powder flow ability, based on Hausner Ratio and Carr Index.

### 3.10. Aerosol Properties of Microspheres

Powder aerosolization of microspheres was tested while using a commercially available cyclohaler-type capsule inhaler (Adamed, Pieńków, Poland). The capsules used in the experiment were loaded with approximately 10 mg powder aliquots. Volumetric particle size distribution was determined using a laser diffraction aerosol spectrometer (Spraytec, Malvern Instruments, Malvern, UK) that was equipped with an inhalation cell. The aerosols were created by the airflow through the inhaler, drawn by the air pump at the rate of 100 dm^3^/min, which is the standard value used during medical inhalations with this type of inhaling device [40,41].

Based on the size distribution of aerosol particles, two essential factors were evaluated: the volumetric median diameter (VMD = *D_V_*50) and the percentage of particle fraction smaller than 5 µm (fine particle fraction, FPF). Both of the parameters are the essential quality indicators of aerosol that is produced by the inhalation drug delivery system [42,43]. In addition, the percentage of particles smaller than 10 µm (inhalable particles) and value of span were determined. Span informs about the width of particle size distribution and it is defined as:

(14) $$Span=\;\frac{D_{V}90-\;D_{V}10}{D_{V}50}$$

where *D_V_*xx denotes xx percentile of the cumulative volumetric particle size distribution.

## 4. Conclusions

In the course of this study, new polyanhydrides that were composed of betulin disuccinate and dicarboxylic derivatives of poly(ethylene glycol) were obtained and characterized. The obtained copolymers undergo hydrolytic degradation to betulin disuccinate and dicarboxylic derivatives of PEG under physiological conditions (37 °C, pH = 7.4). The biological activity of betulin is well documented, and it has been proven to be effective in both oncological [44,45] and bacterial [46] lung diseases. Furthermore, the dicarboxylic derivatives of PEG are approved by FDA for use in drug delivery systems. The use of such polymers in biological systems enables a controlled release of DBB, which is dictated by the degradation rate of the polymer.

Karlina and Pozharitskaya et al. recently investigated some inhalable lactose based nanosystems of betulin delivery [47,48]. They exhibited a successful deposition of betulin in the lower part of the respiratory tract and significantly increased the bioavailability of betulin. This showcased the potential for the viability of betulin based polymer systems in respiratory delivery.

The microspheres that were obtained by us possessed suitable sizes and characteristics for use in inhalation systems, which was also confirmed during aerosolization study in a commercial inhaler. This makes them suitable candidates for use in the treatment of lung diseases. We believe that the administration of betulin in the polymeric form and direct delivery to the lung will increase the substance’s therapeutic potential. Taking betulin’s low toxicity, as well as its protective effects on lung injury [46,49], into account, betulin based polymers look promising as pulmonary drug delivery systems, both when used as the primary active substance or when used in conjunction with other pharmaceutical agents for the treatment of lung diseases.

## Figures and Tables

**Table 1:** Characteristic of polyanhydrides.

Polyanhydride	Feed Ratio DBB:PEG [mol/mol]	DBB:PEG in Polymer [mol/mol] Calculated from ^1^H NMR	Mn (^1^H NMR)	Molecular Weight (GPC)			Tg * [°C] (DSC)
				M_n_	M_w_	DP	
polyDBB	—	—	8200	8500	25,000	2.94	124.0
DBB_PEG_600_20	1:0.27	1:0.19	17,700	1900	4100	2.16	46.3
DBB_PEG_600_40	1:0.71	1:0.59	14,900	1600	2500	1.56	−40.0
DBB_PEG_600_60	1:1.61	1:1.30	56,400	2100	4600	2.19	immeasurable
DBB_PEG_600_80	1:4.29	1:4.19	50,600	—	—	—	immeasurable
DBB_PEG_250_20	1:0.64	1:0.51	8900	1300	1700	1.31	42.0
DBB_PEG_250_40	1:1.71	1:1.66	6000	1500	2100	1.40	−47.4; 23.9
DBB_PEG_250_60	1:3.86	1:3.65	8400	1300	1800	1.38	−41.6; 16.3
DBB_PEG_250_80	1:10.29	1:6.76	9200	—	—	—	−29.5

* Tg—glass temperature of polymers determined as a midpoint of glass transition.

**Table 2:** Solubilities of polyanhydrides.

Polyanhydride	Acetone	H_2_O	EtOH	Toluene	Diethyl Ether	THF	DMSO	CHCl_3_	CH_2_Cl_2_	Hexane
polyDBB	—	—	—	+	—	+	±	+	+	—
polyPEG_250	±	+	+	—	—	—	+	+	+	—
polyPEG_600	+	+	+	+	—	+	+	+	+	—
DBB_PEG_600_20	—	—	—	+	—	+	±	+	+	—
DBB_PEG_600_40	+	—	—	+	—	+	±	+	+	—
DBB_PEG_600_60	+	—	—	+	—	+	+	+	+	—
DBB_PEG_600_80	+	—	±	+	—	+	+	+	+	—
DBB_PEG_250_20	—	—	—	+	—	+	±	+	+	—
DBB_PEG_250_40	+	—	—	+	—	+	+	+	+	—
DBB_PEG_250_60	+	—	—	+	—	+	+	+	+	—
DBB_PEG_250_80	±	—	+	+	—	+	+	+	+	—

+ soluble, ± partially soluble, — insoluble.

**Table 3:** Cytostatic activity of DBB and polyanhydrides against various cancer cell lines as well as a normal control (HDF), expressed as IC_50_^a^.

Compound	Cytostatic Activity IC_50_ [µg/mL]						
	HeLa	MCF-7	A-549	U-87 MG	KB	HepG2	HDF
DBB ^b^	8.25 ± 0.81	7.26 ± 0.79	7.09 ± 0.01	7.37 ± 0.26	7.17 ± 0.93	8.02 ± 0.04	14.80 ± 0.06
polyDBB ^b^	16.23 ± 0.72	13.38 ± 0.06	16.19 ± 0.31	16.07 ± 0.02	17.81 ± 0.03	15.93 ± 0.12	27.13 ± 0.01
PEG_600_20	11.03 ± 0.27	10.33 ± 0.07	11.95 ± 1.03	9.16 ± 0.83	11.76 ± 0.05	11.44 ± 0.49	28.02 ± 0.04
PEG_600_40	9.03 ± 0.51	9.79 ± 0.02	8.62 ± 0.19	9.07 ± 0.09	9.37 ± 0.02	9.84 ± 0.91	17.22 ± 0.88
PEG_250_20	6.22 ± 0.61	—	6.75 ± 0.03	6.62 ± 0.46	6.01 ± 0.77	6.29 ± 0.19	10.55 ± 0.41
PEG_250_60	4.19 ± 0.33	—	4.07 ± 0.05	4.82 ± 0.73	4.21 ± 0.31	4.09 ± 0.35	7.63 ± 0.44
Cytarabine ^c^	1.40 ± 0.08	—	1.17 ± 0.21	1.03 ± 0.25	0.95 ± 0.02	1.49 ± 0.04	1.94 ± 0.01
Actinomycin ^c^	1.13 ± 0.01	—	1.03 ± 0.83	0.92 ± 0.59	1.07 ± 0.05	1.19 ± 0.02	2.28 ± 0.11

^a^*n* = 3 (*t*-test), *p *< 0.05. ^b^ results from our previous work ^c^ Cytarabine and actinomycin D were used as the standard.

**Table 4:** Size and size distribution of microspheres calculated from optical microscope and SEM images.

Polyanhydride	Homogenizer rpm	Optical Microscope (OM)			SEM		
		D_n_ [µm]	S	D_v_/D_n_	D_n_ [µm]	S	D_v_/D_n_
DBB_PEG_250_20	3000	6.12	2.13	1.32	3.6	2.8	2.9
	18,000	3.54	0.89	1.18	1.9	1.2	2.1
DBB_PEG_250_40	3000	14.74	6.64	1.46	7.6	6.7	2.8
	18,000	2.18	0.75	1.34	1.6	0.9	1.7
DBB_PEG_600_20	3000	14.98	5.63	1.38	12.1	6.0	1.6
	9000	7.09	2.80	1.38	5.8	2.9	1.7
	18,000	2.98	0.98	1.34	2.6	1.1	1.5

D_n_—number average diameters, D_v_—volume average diameters, S—standard deviation and D_v_/D_n_—dispersity index.

**Table 5:** Aerodynamic characteristic of microspheres obtained from polyDBB_PEG.

Polyanhydride	Homogenizer rpm	Powder Density [g/cm^3^]		dae ^a^		IC ^b^	HR ^c^
		Untapped	Tapped	OM	SEM		
DBB_PEG_250_20	3000	0.11	0.26	3.12	1.84	55.7	2.26
	18,000	0.10	0.18	1.50	0.81	41.3	1.70
DBB_PEG_250_40	3000	0.12	0.27	7.66	3.95	53.3	2.14
	18,000	0.12	0.25	1.09	0.80	53.3	2.14
DBB_PEG_600_20	3000	0.21	0.31	8.34	6.74	30.9	1.45
	9000	0.18	0.28	3.75	3.07	33.3	1.50
	18,000	0.20	0.27	1.55	1.35	25.0	1.30

^a^ Aerodynamic diameter calculated on the basis of Equation (5); ^b^ Carr’s index (IC) calculated from tapped and untapped density on the basis of Equation (12); ^c^ Hausner Ratio (HR) calculated from tapped and untapped density on the basis of Equation (13).

**Table 6:** Aerosol properties of selected microspheres ^a^ obtained from polyDBB_PEG_600_20.

Parameters	Microspheres	
	9000 rpm	18,000 rpm
VMD (*D_V_*50)	8.17 ± 4.57	6.73 ± 4.81
Span	2.30 ± 4.19	15.62 ± 7.05
%V < 10 µm [%]	62.57 ± 8.40	60.47 ± 16.19
%V < 5 µm [%] (FPF)	19.82 ± 2.81	38.96 ± 10.62

^a^ Microspheres selected based on their aerodynamic characteristics (Table 5).

**Table 7:** Feed ratio of DBB and PEG.

Polyanhydride	Feed Ratio [% *w/w*]		Feed Ratio DBB:PEG [mol/mol]
	DBB	PEG	
polyDBB	100	0	—
DBB_PEG_600_20	80	20	1:0.27
DBB_PEG_600_40	60	40	1:0.71
DBB_PEG_600_60	40	60	1:1.61
DBB_PEG_600_80	20	80	1:4.29
DBB_PEG_250_20	80	20	1:0.64
DBB_PEG_250_40	60	40	1:1.71
DBB_PEG_250_60	40	60	1:3.86
DBB_PEG_250_80	20	80	1:10.29

**Table 8:** Powder flowability based on Hausner Ratio and Carr Index [39].

Flow Character	HR ^b^	IC ^a^
Excellent/very free flow	1.10–1.11	≤10
Good/free flow	1.12–1.18	11–15
Fair	1.19–1.25	16–20
Passable	1.26–1.34	21–25
Poor/cohesive	1.35–1.45	26–31
Very poor	1.46–1.59	32–37
Extremely poor/approx. non-flow	>1.60	>38

^a^ Carr’s index (indicator of the compressibility of a powder, IC) calculated from tapped and untapped density on the basis of Equation (12); ^b^ Hausner Ratio (HR) calculated from tapped and untapped density on the basis of Equation (13).

## Data Availability

The data presented in this study are available on request from the corresponding author.

## Supplementary Materials

The following are available online at https://www.mdpi.com/1422-0067/22/3/1090/s1.
