# Supplementary material for: Bioactive Betulin and PEG Based Polyanhydrides for Use in Drug Delivery Systems
Source: Int J Mol Sci. 2021 Jan 22;22(3):1090. doi: 10.3390/ijms22031090 (PMC7865682; doi:10.3390/ijms22031090)
Supplement: Supplementary file 1 [file ijms-22-01090-s001.pdf]

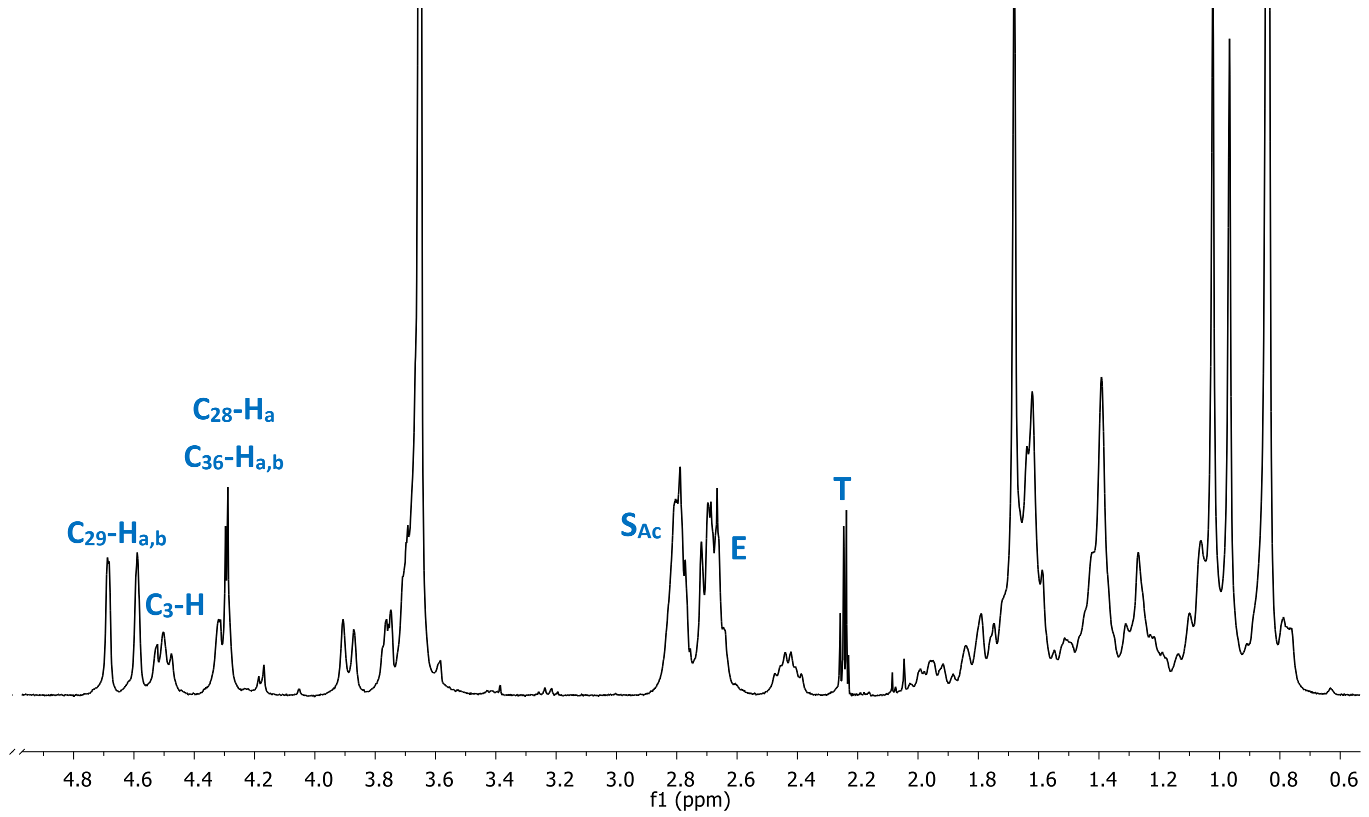

Figure S1. 1H NMR spectrum of polyDBB_PEG

Formulas used to calculate the molecular weight of polymers by [1] H NMR:

$$M_w = n_{DBB}M_{DBB} + n_{PEG}M_{PEG} + M_T$$

$$I_{[1H]DBB} = (I_{C29-Ha} + I_{C29-Hb} + I_{C3-H} + I_{SAc} + I_E)/11$$

$$I_{[1H]T} = I_T/6$$

$$I_{[1H]PEG} = \left(I_{\delta=4.3\,ppm} - I_{[1H]DBB}\right)/4$$

$$n_{DBB} = \frac{I_{[1H]DBB}}{I_{[1H]T}}$$

$$n_{PEG} = \frac{I_{[1H]PEG}}{I_{[1H]T}}$$

where:
M_{DBB} –molar mass of repeating unit of DBB in polyanhydride equal to 642.86g/mol,
M_{PEG} –molar mass of repeating unit of PEG equal to 250 or 600 g/mol,
M_T – molar mass of terminal groups (end groups) in polyanhydride equal to 102 g/mol (molar mass of acetic anhydride),
$I_{[1H]DBB}$ –intensity of one DBB proton,
$I_{[1H]PEG}$ – intensity of one PEG proton,

$I_{[1H]T}$ – intensity of one proton of end groups,
$I_{\delta = 4.30\ ppm}$ – intensity of signal of five protons (C_{28}-H_a and -$CH_2C(O)OC(O)$- in PEG),
I_T – intensity of signal of terminal groups (δ = 2.24 ppm),
$I_{(C29-Ha)}$ and $I_{(C29-Hb)}$ – intensity of signal assigned to methylene protons at the double-bonded carbon (δ = 4.68 and 4.59 ppm),
$I_{(C3-H)}$ – intensity of signal assigned to metine proton in the ring of betulin(δ = 4.50 ppm),
I_{SAc} – intensity of signal assigned to methylene protons in the anhydride moiety (δ = 2.82-2.77 ppm),
I_E - intensity of signal assigned to methylene protons in the ester moiety (δ = 2.74-2.64 ppm).

Intensities of signals used to calculate the molecular weights of the polyanhydrides:

Table S1. Intensities of signals used to calculate the molecular weights of DBB_PEG_250_20

Signal	Chemical shift δ [ppm]	Intensity	Assigned protons
C_3-H	4.50	1.52	~C**H**(-OCO)~
C_{28}-H_a and C_{36}-$H_{a,b}$	4.30	4.75	~C-C**H₂**-O-CO-CH₂~ and -C**H₂**-C(O)OC(O)~
C_{29}-H_a	4,68	1.30	**H₂C**=C(-CH₃)-CH~
C_{29}-H_b	4,59	1.40	
SAc	2.82-2.77	5.76	-OCOOC-C**H₂**-CH₂-COO-
E	2.74-2.64	6.33	-OCOOC-CH₂-C**H₂**-COO-
T	2.24	0.77	end groups

Table S2. Intensities of signals used to calculate the molecular weights of DBB_PEG_250_40

Signal	Chemical shift δ [ppm]	Intensity	Assigned protons
C_3-H	4.50	1.12	~C**H**(-OCO)~
C_{28}-H_a and C_{36}-$H_{a,b}$	4.30	9.22	~C-C**H₂**-O-CO-CH₂~ and -C**H₂**-C(O)OC(O)~
C_{29}-H_a	4,68	0.83	**H₂C**=C(-CH₃)-CH~
C_{29}-H_b	4,59	1.04	
SAc	2.82-2.77	4.82	-OCOOC-C**H₂**-CH₂-COO-
E	2.74-2.64	5.30	-OCOOC-CH₂-C**H₂**-COO-
T	2.24	1.26	end groups

Table S3. Intensities of signals used to calculate the molecular weights of DBB_PEG_250_60

Signal	Chemical shift δ [ppm]	Intensity	Assigned protons
C_3-H	4.50	0.76	~C**H**(-OCO)~
C_{28}-H_a and C_{36}-$H_{a,b}$	4.30	14.79	~C-C**H₂**-O-CO-CH₂~ and -C**H₂**-C(O)OC(O)~
C_{29}-H_a	4,68	0.54	**H₂C**=C(-CH₃)-CH~
C_{29}-H_b	4,59	0.67	
SAc	2.82-2.77	3.79	-OCOOC-C**H₂**-CH₂-COO-
E	2.74-2.64	4.22	-OCOOC-CH₂-C**H₂**-COO-
T	2.24	1.04	end groups

Table S4. Intensities of signals used to calculate the molecular weights of DBB_PEG_250_80

Signal	Chemical shift δ [ppm]	Intensity	Assigned protons
C_3-H	4.50	0.80	~C**H**(-OCO)~
C_{28}-H_a and C_{36}-$H_{a,b}$	4.30	19.61	~C-C$\mathbf{H_2}$-O-CO-CH_2~ and -C$\mathbf{H_2}$-C(O)OC(O)~
C_{29}-H_a	4,68	0.75	$\mathbf{H_2C}$=C(-CH_3)-CH~
C_{29}-H_b	4,59	0.76	
SAc	2.82-2.77	2.64	-OCOOC-C$\mathbf{H_2}$-CH_2-COO-
E	2.74-2.64	2.75	-OCOOC-CH_2-C$\mathbf{H_2}$-COO-
T	2.24	1.07	end groups

Table S5. Intensities of signals used to calculate the molecular weights of DBB_PEG_600_20

Signal	Chemical shift δ [ppm]	Intensity	Assigned protons
C_3-H	4.50	1.25	~C**H**(-OCO)~
C_{28}-H_a and C_{36}-$H_{a,b}$	4.30	2.44	~C-C$\mathbf{H_2}$-O-CO-CH_2~ and -C$\mathbf{H_2}$-C(O)OC(O)~
C_{29}-H_a	4,68	1.16	$\mathbf{H_2C}$=C(-CH_3)-CH~
C_{29}-H_b	4,59	1.27	
SAc	2.82-2.77	4.85	-OCOOC-C$\mathbf{H_2}$-CH_2-COO-
E	2.74-2.64	6.77	-OCOOC-CH_2-C$\mathbf{H_2}$-COO-
T	2.24	0.35	end groups

Table S6. Intensities of signals used to calculate the molecular weights of DBB_PEG_600_40

Signal	Chemical shift δ [ppm]	Intensity	Assigned protons
C_3-H	4.50	0.91	~C**H**(-OCO)~
C_{28}-H_a and C_{36}-$H_{a,b}$	4.30	3.48	~C-C$\mathbf{H_2}$-O-CO-CH_2~ and -C$\mathbf{H_2}$-C(O)OC(O)~
C_{29}-H_a	4,68	0.76	$\mathbf{H_2C}$=C(-CH_3)-CH~
C_{29}-H_b	4,59	0.85	
SAc	2.82-2.77	4.24	-OCOOC-C$\mathbf{H_2}$-CH_2-COO-
E	2.74-2.64	4.67	-OCOOC-CH_2-C$\mathbf{H_2}$-COO-
T	2.24	0.41	end groups

Table S7. Intensities of signals used to calculate the molecular weights of DBB_PEG_600_60

Signal	Chemical shift δ [ppm]	Intensity	Assigned protons
C_3-H	4.50	0.85	~C**H**(-OCO)~
C_{28}-H_a and C_{36}-$H_{a,b}$	4.30	4.92	~C-C$\mathbf{H_2}$-O-CO-CH_2~ and -C$\mathbf{H_2}$-C(O)OC(O)~
C_{29}-H_a	4,68	0.73	$\mathbf{H_2C}$=C(-CH_3)-CH~
C_{29}-H_b	4,59	0.74	
SAc	2.82-2.77	2.95	-OCOOC-C$\mathbf{H_2}$-CH_2-COO-
E	2.74-2.64	3.41	-OCOOC-CH_2-C$\mathbf{H_2}$-COO-
T	2.24	0.81	end groups

Table S8. Intensities of signals used to calculate the molecular weights of DBB_PEG_600_80

**Signal**	**Chemical shift δ [ppm]**	**Intensity**	**Assigned protons**
C_3-H	4.50	0.12	~C**H**(-OCO)~
C_{28}-H_a and C_{36}-$H_{a,b}$	4.30	2.83	~C-C**H**$_2$-O-CO-CH$_2$~ and -C**H$_2$**-C(O)OC(O)~
C_{29}-H_a	4,68	0.12	**H$_2$C**=C(-CH$_3$)-CH~
C_{29}-H_b	4,59	0.13	
SAc	2.82-2.77	0.59	-OCOOC-C**H$_2$**-CH$_2$-COO-
E	2.74-2.64	0.78	-OCOOC-CH$_2$-C**H$_2$**-COO-
T	2.24	0.06	end groups
